# Stress and Inflammatory Bowel Disease: Clear Mind, Happy Colon

**DOI:** 10.7759/cureus.25006

**Published:** 2022-05-15

**Authors:** Joaquim Francisco Maria De Sousa, Smit Paghdar, Taheseen M Khan, Nishant P Patel, Savitri Chandrasekaran, Nicholas Tsouklidis

**Affiliations:** 1 Internal Medicine, California Institute of Behavioral Neurosciences & Psychology, Fairfield, USA; 2 Surgery, S.S. Institute of Medical Sciences and Research Centre, Davanagere, IND; 3 Emergency Medicine, Healthway Hospital, Goa, IND; 4 Internal Medicine, Surat Municipal Institute of Medical Education and Research (SMIMER), Surat, IND; 5 General Practice, Mahatma Gandhi Mission Medical College, Navi Mumbai, IND; 6 Psychiatry, California Institute of Behavioral Neurosciences & Psychology, Fairfield, USA; 7 Internal Medicine, Government Medical College, Surat, Surat, IND; 8 General Practice, Indira Gandhi Medical College and Research Institute, Pondicherry, IND; 9 Pediatrics, Wyckoff Heights Medical Center, Brooklyn, USA; 10 Health Care Administration, University of Cincinnati Health, Cincinnati, USA; 11 Medicine, California Institute of Behavioral Neurosciences & Psychology, Fairfield, USA; 12 Medicine, Atlantic University School of Medicine, Gros Islet, LCA

**Keywords:** crohn's disease, mental health, ulcerative colitis, uc, gut-brain axis, psychological stress, ibd

## Abstract

Inflammatory bowel disease (IBD) is a condition whose prevalence in the general population worldwide is increasing at an exponential pace. Many risk factors affect the incidence, progression, and overall outcome of IBD, one of them being psychological stress. This study examined the relationship between psychological stress and inflammatory bowel disease.

A search for relevant studies was conducted using PubMed, Google Scholar, ResearchGate, and SCOPUS. A systematic review was conducted on the relevant articles after critical appraisal.

This article mainly focused on studies that evaluated the presence of inflammatory markers observed in individuals who have been diagnosed with IBD and have high levels of psychological stress. It also assessed if lowering an individual’s psychological stress could help improve the outcomes of IBD.

Psychological stress can have a detrimental effect on individuals diagnosed with IBD. There is a need to conduct studies that can further confirm the association between psychological stressors, mental health conditions, and IBD. We should also encourage medical practitioners to educate patients who have been diagnosed with IBD regarding the benefits of stress reduction.

## Introduction and background

Inflammatory bowel disease (IBD) is an umbrella term that encompasses a group of inflammatory conditions that affect the small intestine and the colon. Crohn’s disease (CD) and ulcerative colitis (UC) are the two main subtypes of IBD [1].

UC is a chronic, inflammatory condition often limited to the colon and the rectum [2]. Patients with UC typically present with bloody diarrhea, tenesmus, and abdominal pain [3]. This was the first subtype of IBD to be discovered and serves as a starting point in the history of IBD. However, it is highly likely that the other forms were also present but not fully understood yet [4]. UC was first described in 1875 by two English physicians, Wilks and Moxon, who helped differentiate it from other diarrheal diseases caused by infectious agents [5]. In the following decades, the overall understanding of UC grew tremendously. Its role in causing cancer was better understood, and multiple treatment modalities followed soon after that.

CD is defined as “a chronic transmural inflammatory bowel disease, with skip lesions that may involve any part of the GI tract from the mouth to anus” [6]. Giovanni Battista Morgagni first described this condition. One of his case reports discussed a 20-year-old male patient who died following a prolonged spell of illness, including fever, abdominal pain, and bloody diarrhea. An autopsy was later performed, revealing perforations and transmural inflammation with ulceration, which extended from the terminal ileum to the colon. The report also suggested the presence of mesenteric lymphadenopathy and splenomegaly. Though Morgagni’s work was essential to introducing CD, it was an article written by Burrill B. Crohn, Leon Ginzburg, and Gordon D. Oppenheimer published in the *Journal of the American Medical Association* that gave us a better understanding of CD. The authors described a condition that affected the terminal part of the ileum. Its pathology included the presence of granulomas along with chronic necrotizing and cicatrizing inflammation in the ileum. There was also the presence of ulceration in the intestinal mucosa and connective tissue reactions, which repeatedly occurred, resulting in the obstruction of the intestinal lumen leading to the formation of fistulas. CD was recognized as a separate entity from UC in 1932 by Crohn et al. [4].

The prevalence rate of IBD in America increased from 0.3% in the early 2000s and is projected to cross 1.3% this decade. Due to the increasing number of newly diagnosed cases, there has also been a new interest in deciphering the risk factors that might be responsible for causing this disease [7]. Major risk factors such as high-fat diet, smoking (a risk factor for CD and a protective factor for UC), psychological stress, and appendectomy (a protective factor for UC and a risk factor for CD) have all been attributed to increasing the chances of being diagnosed with IBD in the future [8].

The relationship between the immune system, nervous system, and psychological processes is being established and has always been an area of considerable interest [9]. There is mounting evidence that associations between these systems play an essential role in IBD [10,11]. Psychological stressors affect the gut through various mechanisms, such as increased production of pro-inflammatory cytokines, activation of macrophages, and tumor necrosis factor through the hypothalamus-pituitary-adrenal axis [12].

IBD and psychological disorders share multiple pro-inflammatory pathways, such as the clinical expression of activated immune-inflammatory, oxidative, and nitrosative stress (IO&NS) pathways, including tryptophan catabolite (TRYCAT), autoimmune, and gut-brain pathways. These shared pathways are involved in the pathogenesis of IBD and psychological disorders, which can explain the concomitant flare-up of IBD in patients with depression as well as the worsening treatment outcomes of IBD patients who are diagnosed with psychological disorders [13].

**Figure 1:**
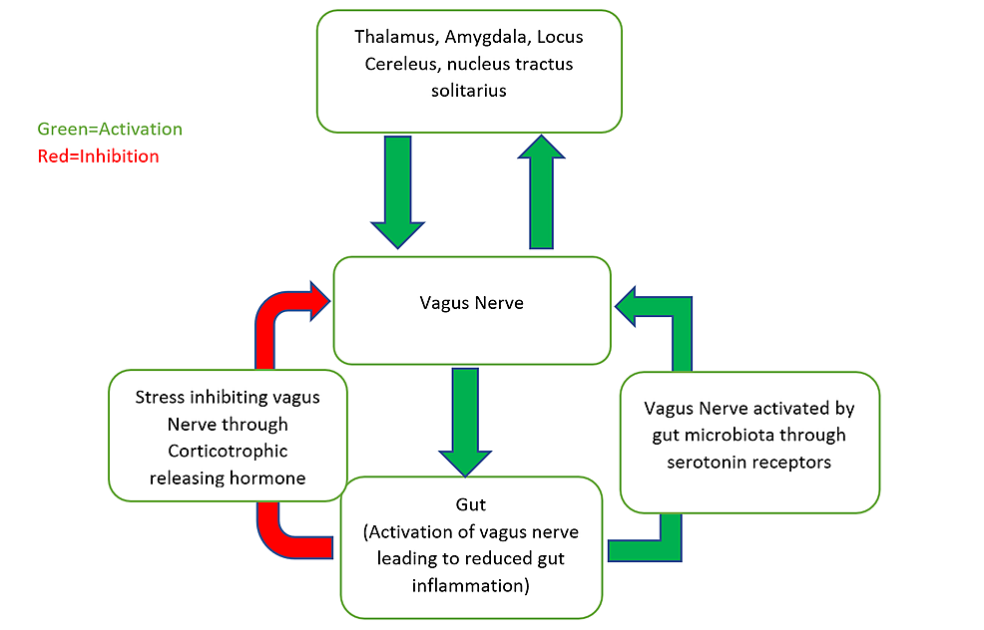
Pathogenesis of IBD through the gut-brain axis. IBD: inflammatory bowel disease

In addition to the above-mentioned shared inflammatory pathways, the gut-brain axis has also been mentioned as one of the shared pathways between IBD and psychological disorders. This pathway involves bidirectional communication between the gut and the central nervous system. The principal component of this is the autonomic nervous system, of which the vagus nerve forms the core component. Stress is known to inhibit vagal nerve stimulation, which has anti-inflammatory properties, resulting in deleterious effects on the gastrointestinal tract. Early recognition and efforts to lower stress levels should help [14]. Figure 1 demonstrates the pathogenesis of IBD through the gut-brain axis.

The United States had the highest age-standardized prevalence rate (464.5 [438.6-490.9]) per 100,000 population), followed by the United Kingdom (449.6 [420.6-481.6] per 100,000) [15]. As with any highly prevalent disease, there is a need to know the factors that trigger this condition. Psychological stress was a risk factor in many of these patients. It was also found that psychological stress caused a worsening of the quality of life in some IBD patients, and reducing stress levels in IBD patients helped in their remission [16]. Psychological stress is a risk factor that can be decreased if detected. Over time, various methods have been shown to help reduce stress, ranging from coping strategies to medications and lifestyle changes. Hence, gaining a better understanding of how psychological stress impacts the incidence and the outcomes of IBD has become paramount.

This review aims to explore the relationship between psychological stress on the incidence and outcomes of IBD. Further, this article aims to evaluate if reducing stress can help improve the chances of remission of IBD.

This article was previously presented as a poster at the International Virtual Medical Conference (IVMC): Spring Conference on April 23, 2022.

## Review

Methodology

PubMed, Medline, SCOPUS, Cochrane Library, and Google Scholar were exclusively and systematically searched to collect relevant data. Studies that explored the relationship between psychological stress and the onset and progression of IBD were selected. Before screening and applying any inclusion/exclusion criteria, 706 articles were found on PubMed. Once inclusion criteria were applied, relevant studies included: (1) those published in English, (2) published within the last 27 years, (3) human studies, and (4) sample sizes of more than 20. Articles excluded from this review included those with sample sizes of less than 20 subjects and those containing animal studies. Then, a critical appraisal was performed using the Assessment of Multiple Systematic Reviews (AMSTAR) checklist. Eight studies were selected for this review after applying inclusion criteria and performing quality checks.

Results

Eight studies were selected to evaluate the effect of psychological stress on the onset, progression, and IBD outcomes. Two studies reported that psychological stress can cause an increase in inflammatory markers such as leucocyte number, interleukin (IL) 6 and IL-13 levels, tumor necrosis factor (TNF)-alpha, and natural killer (NK) cell numbers [17,18]. All of these inflammatory cells are generally raised in IBD. Two other studies showed that stress reduction exercises helped reduce the symptoms seen during IBD, improving patients’ overall health [19,20]. Three studies showed that stress was responsible for relapses in patients with IBD, and patients exposed to stressful events were more likely to experience a relapse than those who had less stressful lives [21-23]. In contrast, one study reported otherwise [24]. Table 1 presents the studies that met the inclusion criteria and the conclusions drawn from them.

**Table 1:** Description of selected studies that met the inclusion criteria for this review. IBD: inflammatory bowel disease; IBS: irritable bowel syndrome; MDD: major depressive disorder; TNF: tumor necrosis factor; UC: ulcerative colitis

Study	Location of study	Study period	Sample size	General conclusions
Tuglu et al. [17]	Turkey	2003	26 MDD patients and 17 control volunteers	On admission, serum TNF-alpha and leukocyte count were significantly higher in MDD patients compared to controls
Mawdsley et al. [18]	United Kingdom	2005	25 patients with inactive UC and 11 healthy volunteers underwent an experimental stress test. In addition, 10 patients with UC and 11 healthy volunteers underwent a control procedure	Acute psychologic stress induces systemic and mucosal pro-inflammatory responses, which could contribute to exacerbations of UC in ordinary life
Kuo et al. [19]	United States	2009–2011	19 IBS and 29 IBD patients	Pain Catastrophizing Scale scores improved significantly post-stress reduction for IBD patients
Gerbarg et al. [20]	United States	2015	29 participants with IBD	Patients with IBD showed improved symptoms after practicing relaxation exercises showed improvement in psychological, physical, and C-reactive protein levels
Persoons et al. [21]	Belgium	2003	100 patients	MDD was a risk factor for failure of remission while on treatment and was responsible for earlier relapses
Bitton et al. [22]	Canada	1998–2002	101 patients	Patients with low stress were less likely to have a relapse of Crohn’s disease
Bernstein et al. [23]	Canada	2010–2011	704 participants	Patients who experienced stressful events were more likely to have flare-ups of IBD
Boye et al. [24]	Norway and Germany	2011	58 patients with UC and 56 patients with Crohn’s disease	Psychological stress management psychotherapy does not improve disease course or reduce the chances of a relapse

Discussion

Effect of Psychological Stress on the Onset of IBD

Psychological stress can have a detrimental effect on our mental health and harm our physical well-being. In a study conducted in 2003 by Tuglu et al., 43 participants were chosen [17]. In total, 26 patients were diagnosed with major depressive disorder, and the remaining 17 were control subjects. This study aimed to examine the relationship between a psychological stressor such as major depressive disorder and an increase in pro-inflammatory cells such as IL-1, IL-6, and TNF-alpha. The measurement of these inflammatory markers was done at the start of the study and after six weeks of anti-depressant treatment. This study confirmed that psychological stressors have a role in increasing inflammatory markers as they were significantly higher in patients with major depressive disorders than in the control, and the levels of inflammatory markers markedly reduced after six weeks of anti-depressive treatment.

Another study by Mawdsley et al. aimed to study the rectal mucosa’s inflammatory response to psychological stress [18]. To assess this, serum IL-6 and IL-13 concentrations, TNF-alpha, and IL-6 production by lipopolysaccharide (LPS)-stimulated whole blood, leukocyte count, NK cell numbers, platelet activation, platelet-leukocyte aggregate (PLA) formation, substance P release, reactive oxygen metabolite (ROM) production, mucosal blood flow (RMBF), and histology were all actively measured in two groups of patients. One group underwent a stress test in which participants were asked to complete a 60-minute intelligent quotient (IQ) test in 50 minutes with music playing in the background and being repeatedly reminded to increase their efforts in completing the test. The second group underwent a control procedure and was asked to complete the same IQ test in 50 minutes but with background music of their choice. The inflammatory markers were measured before and after the process. This study also confirmed the presence of inflammatory response in individuals who experienced psychological stressors, which resulted in their relapses, further confirming that psychological stressors can cause an increase in inflammatory markers, which are seen in IBD. *Effect of Psychological Stress on the Disease Progression in IBD*

Being diagnosed with IBD can be a traumatic experience for individuals from a physical and psychological standpoint. Patients diagnosed with IBD can present with various symptoms, such as abdominal pain, recurring bloody diarrhea, weight loss, and extreme tiredness. Many of these patients suffer from psychological stress due to being diagnosed with a chronic condition and from the symptoms they suffer. A few studies have shown that by reducing psychological stress, a patient’s quality of life is improved as a result of minimizing their psychological stress. One such study by Kuo et al. aimed to investigate the effect of stress reduction techniques on patients diagnosed with IBS and irritable bowel syndrome (IBS) [19]. This study enrolled 19 patients diagnosed with IBS and 29 IBD patients in a nine-week relaxation exercise called relaxation response-based mind-body group intervention (RR-MBI). A questionnaire regarding patients’ symptoms and inflammatory markers was used in a pre-post intervention study and after a short-term follow-up. This study showed that the patients’ pain scores and overall quality of life improved after the relaxation exercise.

Finally, a study by Gerbarg et al. evaluated the effect of a breath-body-mind workshop (a type of relaxation exercise) on various aspects of a patient who was diagnosed with IBD, such as their physical symptoms, inflammatory markers, and their psychological well-being [20]. In total, 29 patients diagnosed with IBD and enrolled in the Jill Roberts IBD centre were chosen and were randomly allocated to a group that would participate in the breath-body-mind workshop or an educational seminar. The IBD questionnaire, fecal calprotectin, C-reactive protein, perceived stress questionnaire, perceived disability scale, Beck anxiety inventory, and Beck depression inventory measures were obtained at baseline to measure the study outcomes and at weeks six and twenty-six. This study showed that patients enrolled in the breath-body-mind workshop were associated with significant improvements in their overall physical symptoms and psychological well-being and showed a drop in their C-reactive protein level. The studies mentioned above showed that stress reduction could benefit patients who have been diagnosed with IBD regarding their physical symptoms and psychological well-being and help improve their overall quality of life. *Effect of Psychological Stress and Mental Health Conditions on the Outcomes of IBD*

Psychological stress and mental health conditions can also affect the disease outcomes, as described in a study by Persoons et al. in Belgium, whose aim was to study the effect of major depressive disorder on the outcomes of patients who were taking infliximab for luminal CD [21]. Major depressive disorder was diagnosed using the Patient Health Questionnaire. A total of 100 consecutive unselected patients were chosen for this prospective study. The patients were assessed at baseline and at four weeks after being treated with infliximab. Assessments included the patients’ clinical, psychosocial, and demographical disease-related biological parameters. The patients were then followed up clinically for the next nine months or had a flare-up. This study pointed out major depressive disorder as a risk factor for treatment failure with patients on infliximab and was responsible for earlier relapses in patients with CD.

A study by Bitton et al. aimed to identify if psychosocial, biological, and clinical parameters could predict relapse in inactive CD [22]. A total of 101 patients were recruited for this study, of whom 14 patients withdrew. Serum cytokines, anti-*Saccharomyces cerevisiae* antibodies, C-reactive protein, erythrocyte sedimentation rate, and intestinal permeability were measured every three months. Psychological distress was measured every month for a year. This study pointed out that patients under low stress or who did not engage in social diversion were less likely to relapse.

A large study conducted by Bernstein et al. aimed to study if stress, infections, antibiotics, or non-steroidal anti-inflammatory drugs trigger symptomatic flares in patients diagnosed with IBD [23]. A flare-up of the disease was identified using the Manitoba Inflammatory Bowel disease index. In total, 704 participants completed a baseline survey. These participants were selected from a population-based IBD register. They were followed up every three months for a year. This survey tracked the use of non-steroidal anti-inflammatory drugs, antibiotics, infections, major life events, and any forms of stressors faced by the participants. This study pointed out that stress was responsible for most symptomatic flare-ups in IBD.

Lastly, a study by Boye et al. aimed to test the theory that psychotherapy helped improve the overall disease progression and prevent relapses in patients diagnosed with IBD [24]. In total, 58 patients with UC and 56 patients with CD were selected and randomized to either receive treatment with standard medical management in one group or receive medical management and psychotherapy in the second group and were assessed at baseline, three, six, twelve, and eighteen months using the Inflammatory Bowel Disease Questionnaire to measure the outcomes. The study concluded that psychotherapy did not help improve the disease progression in patients diagnosed with IBD, nor did it reduce the chances of preventing relapse of the disease. Some of the studies mentioned above highlight that psychological stress and mental health conditions can contribute to relapses and symptomatic flare-ups in patients who have been diagnosed with IBD. Although some above-mentioned studies contradict this fact, this is an area that should be further investigated in the future. It can aid clinicians in formulating a follow-up plan that can help prevent relapses in patients diagnosed with IBD.

Limitations and future recommendations

Several studies and clinical trials did not have a large enough sample size. To better understand the relationship between psychological stress and IBD, much larger sample sizes would be needed in the future. Moreover, a few of the trials that were used are more than 15 years old. We need more studies in the future to confirm the extent to which psychological stress influences the disease course in IBD.

Recommendations for the future include allocating more resources toward further studying the link between IBD and psychological stressors and incorporating stress reduction techniques in the management plan for patients diagnosed with IBD.

## Conclusions

While many risk factors can affect the overall course of disease for a patient who is yet to be diagnosed or has already been diagnosed with IBD, studies have found that stress and mental health conditions can increase inflammatory markers, thus increasing the probability of being diagnosed with IBD in the future. Studies have reported that stress can cause earlier relapses in patients with inactive IBD. In addition to affecting the incidence of IBD, stress reduction can reduce symptoms in patients who have already been diagnosed with IBD, hence improving the quality of their lives. We believe this article can further incite interest in investigating the relationship between IBD and psychological stressors. It can encourage medical practitioners to gauge stress levels in patients diagnosed with IBD during their consultation or with the help of questionnaires. They can then advise these patients of the benefits of stress reduction and recommend ways to do this using techniques such as mindfulness-based interventions, cognitive behavioral therapy, and similar stress alleviating processes.
